# Advances in Genes Associated with Straw Degradation in Rice

**DOI:** 10.3390/life16091491

**Published:** 2026-09-06

**Authors:** Tao Tong, Yangyan Wei, Qingxia Wang, Fanrong Zeng, Yuting Hu, Shuzhen Ye, Zhijuan Ji, Yanli Wang, Shunan Zheng, Younan Ouyang

**Affiliations:** 1 State Key Laboratory for Rice Biology and Breeding, China National Rice Research Institute, Hangzhou 311401, China; tongtao@caas.cn (T.T.); jizhijuan@caas.cn (Z.J.); 2 Northeast Asia Agricultural Science and Technology Innovation Center, Chinese Academy of Agricultural Sciences, Baoqing 155600, China; wangqingxia@caas.cn (Q.W.); 2021720799@yangtzeu.edu.cn (Y.H.); yeshuzhen@caas.cn (S.Y.); 3 Zhejiang Shaoxing Shangyu Agricultural Technique Spreading Center, Shaoxing 312300, China; 15268451035@139.com; 4 Research and Development Center of Rice Cropping Technology, China National Rice Research Institute, Hangzhou 311401, China; 5 MARA Key Laboratory of Sustainable Crop Production in the Middle Reaches of the Yangtze River (Co-Construction by Ministry and Province), College of Agriculture, Yangtze University, Jingzhou 434025, China; fanrong.zeng@yangtzeu.edu.cn; 6 State Key Laboratory for Quality and Safety of Agro-Products, Key Laboratory of Agricultural Microbiome of Zhejiang Province, Key Laboratory of Biotechnology in Plant Protection of MARA, Institute of Plant Protection and Microbiology, Zhejiang Academy of Agricultural Sciences, Hangzhou 310021, China; wangyl@zaas.ac.cn; 7 Rural Energy and Environment Agency, Ministry of Agriculture and Rural Affairs, Beijing 100125, China

**Keywords:** rice, straw degradability, cell wall, cellulose synthase, lignin biosynthesis, brittle culm mutant, hemicellulose modification, gene editing

## Abstract

Rice (*Oryza sativa* L.) generates approximately 300 million tons of straw annually worldwide, and efficient degradation and valorization remain critical bottlenecks for sustainable agriculture. Straw degradability is fundamentally constrained by cell wall recalcitrance, which is determined by the composition and architecture of cellulose, hemicellulose, and lignin, each under genetic regulation. This review proposes a three-parameter analytical framework, including cellulose crystallinity, hemicellulose side-chain modification, and lignin monomer composition and cross-linking density, to systematically assess the regulatory role of endogenous rice genes in straw degradability. We provide a comprehensive synthesis of recent advances in cellulose synthase genes and brittle culm mutants, lignin biosynthesis, cell wall modification genes, and the integration of genetic mapping with molecular breeding strategies. Particular attention is given to the trade-offs between enhanced degradability and agronomic performance, and to emerging strategies, including semi-dominant alleles, tissue-specific promoters, and multi-gene pyramiding, which hold potential for resolving these trade-offs. We conclude by identifying key research gaps and proposing future directions for developing dual-purpose rice cultivars with both high grain yield and superior straw degradability.

## 1. Introduction

Rice (*Oryza sativa* L.) is a crucial staple crop worldwide, generating approximately 300 million tons of straw annually [1]. Traditionally, substantial amounts of rice straw have been eliminated through open-field burning, causing severe air pollution and carbon resource waste. The valorization of rice straw, whether for biofuel production, ruminant feed, or field return as organic amendment, constitutes a significant challenge for sustainable agriculture [2].

The degradation efficiency of rice straw is fundamentally constrained by cell wall recalcitrance, a multi-scale physical and chemical barrier resulting from the intricate interplay among cellulose, hemicellulose, and lignin. The cell wall of rice straw is primarily composed of cellulose (30–47%), hemicellulose (19–35%), and lignin (5–24%), along with significant silica content. Cellulose consists of β-1,4-glucan chains arranged through intermolecular hydrogen bonds to form microfibrils; its crystallinity index (CrI) is the primary physical parameter determining cellulase accessibility [3]. In grasses, hemicellulose is dominated by arabinoxylan (AX), characterized by arabinose side chains which are ester-linked to ferulic acid (FA). These side chains can undergo oxidative coupling to form diferulate cross-links, creating a dense network that encapsulates cellulose microfibrils [4]. Lignin, composed of H (p-hydroxyphenyl), G (guaiacyl), and S (syringyl) units, constitutes the ultimate chemical barrier by facilitating non-specific enzyme adsorption and physical encapsulation of polysaccharides [5].

**Figure 1:**
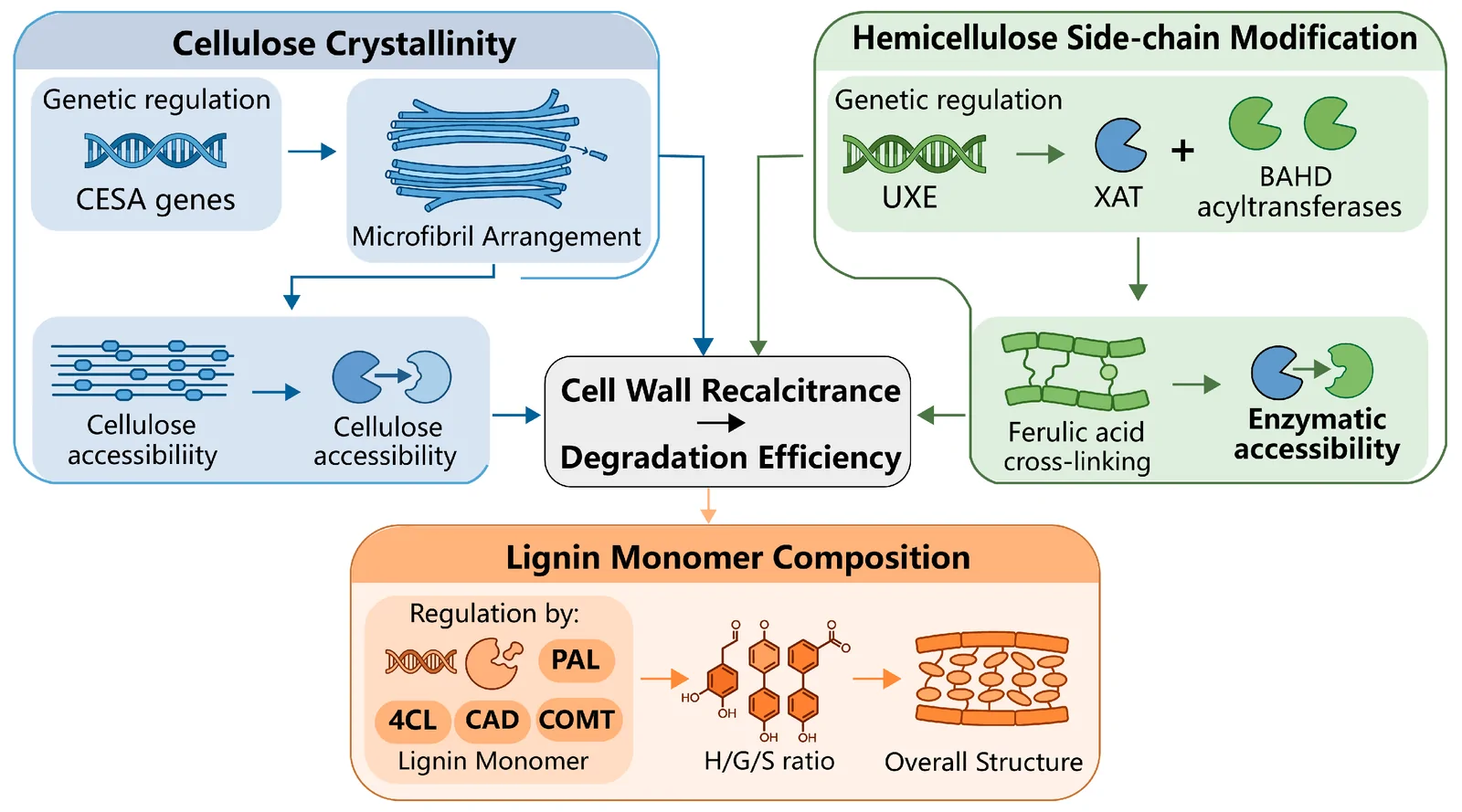
Three-parameter analytical framework for rice straw degradability. The framework evaluates straw degradability through three core dimensions: (1) cellulose crystallinity, determined by *CESA* family genes (*OsCESA4*, *OsCESA7*, and *OsCESA9*), which governs the crystallinity index (CrI) and cellulase accessibility of β-1,4-glucan microfibrils; (2) hemicellulose side-chain modification, regulated by arabinoxylan substitution genes (*UXE*, *XAT*, and *BAHD* acyltransferases) and ferulate metabolism genes (*OsHCALDH2*/*3*), which controls the density of diferulate cross-links encapsulating cellulose microfibrils; and (3) lignin monomer composition and cross-linking density, shaped by phenylpropanoid pathway genes (*OsC3′H1*, *OsCAD2*, *OsCOMT*, and *OsFNSII*), which determines the H/G/S ratio, tricin nucleation, and lignin-carbohydrate complex (LCC) formation. These three parameters collectively define the multi-scale recalcitrance of the rice cell wall, providing a systematic basis for evaluating how endogenous genes regulate straw degradability.

Conventional physicochemical pretreatment methods, such as acid hydrolysis, alkaline treatment, and steam explosion, can partially disrupt cell wall integrity. However, these methods are energy-intensive, produce inhibitory by-products, and are economically unfeasible for large-scale applications. As a result, a paradigm shift has emerged, focusing on the genetic modification of rice cell wall biosynthesis to develop cultivars with intrinsically lower biomass recalcitrance, thereby reducing the necessity for severe pretreatment [6]. Before presenting the framework, it is essential to define the scope of this review. The term ‘genes associated with straw degradation in rice’ refers specifically to endogenous rice genes that regulate the composition and architecture of the cell wall of mature straw, thereby determining its intrinsic degradability. This review does not address the genes of microbial communities that decompose rice straw after harvest. However, the interaction between rice genotype and microbial ecology is discussed where relevant. Rather than cataloguing reported genes, this review aims to provide a critical synthesis that distinguishes well-validated targets from preliminary associations. It also evaluates the consistency of reported effects across genotypes and experimental conditions, and identifies which traits exert the greatest influence on straw recalcitrance. In this review, we propose a three-parameter analytical framework-cellulose crystallinity, hemicellulose side-chain modification, and lignin monomer composition and cross-linking density, which serves as the core dimensions for systematically evaluating the regulation of straw degradability by endogenous rice genes (Figure 1).

Although silica constitutes a significant component of rice straw cell walls (typically 5–15% of dry weight) and exhibits a well-documented negative correlation with digestibility, it is not incorporated as a core parameter in the current framework for two reasons. Firstly, silica deposition in rice is predominantly governed by environmental factors rather than by the kind of biosynthetic pathway genes that regulate cellulose, hemicellulose and lignin. Silicon transport genes (e.g., *OsLsi1* and *OsLsi2*) control uptake and distribution but do not participate directly in cell wall polymer biosynthesis. Secondly, silica content varies substantially across growing conditions and soil types, making it less genetically tractable as a breeding target than the three polymer-based parameters. Nevertheless, silica is discussed in Section 4.1 as a complementary factor that should be considered alongside the three-parameter framework, particularly for rice genotypes cultivated in environments with high silicon content.

As shown in Table 1, the three principal components of the cell wall demonstrate a distinct progressive relationship: cellulose crystallinity determines the baseline level of enzyme accessibility, hemicellulose side-chain cross-linking regulates cell wall porosity, and lignin encapsulation constitutes the ultimate physicochemical barrier. It is imperative to clarify from the outset that rice genes associated with straw degradability are genes that govern the recalcitrance of the cell wall in mature straw, rather than genes that encode enzymatic systems capable of independently degrading straw. In natural field environments, straw decomposition is driven primarily by indigenous or exogenous microbial communities. Rice genotypes provide the substrate accessibility background that determines how readily these communities can act [7]. This distinction has important implications for interpreting experimental results, as enzymatic saccharification improvements cannot be directly equated with field decomposition improvements.

## 2. Cellulose Synthase Genes and Brittle Culm Mutants

Brittle culm (bc) mutants serve as crucial genetic resources for investigating the mechanisms underlying cellulose synthesis and straw degradability in rice. The secondary wall cellulose synthase complex (CSC), characterized by a plasma membrane-localized rosette structure with six-fold symmetry, is composed of three catalytic subunits: OsCESA4, OsCESA7, and OsCESA9. Null mutations in any of these genes result in drastically reduced cellulose content, severely deformed secondary cell walls, and brittle culm phenotypes, confirming their essential and non-redundant roles in secondary wall cellulose synthesis [8].

A significant finding from studies on cellulose synthase (CESA) mutants is that modifications to the cell wall initiate systemic reprogramming rather than eliciting linear responses at the level of individual genes. Li et al. (2018) showed that the *FC17*/*CESA4* mutation not only reduces cellulose content and crystallinity but also induces compensatory increases in hemicellulose and lignin [9]. This finding suggests that the cell wall maintains homeostatic balance through coordinated adjustments among its three major components. Despite this compensation, the *FC17* mutation increased biomass saccharification efficiency by 1.68-fold and simultaneously enhanced lodging resistance, demonstrating that specific *CESA* alleles can enhance both degradability and mechanical strength. Early feed evaluation of *OsCESA4* mutants further showed that dry matter, neutral detergent fiber (NDF), cellulose, and hemicellulose disappearance rates were higher at 24 and 48 h, with effective dry matter digestibility increasing from 26.7% to 31.4% [10]. However, as emphasized by Elicit analyses, this evidence from ruminant feed evaluation should not be directly extrapolated to predict anaerobic decomposition rates in paddy fields. These two degradation processes involve different microbial communities, time scales, and environmental conditions. Importantly, cell wall components do not function independently. The *FC17*/*CESA4* example shows that reducing cellulose crystallinity leads to compensatory increases in hemicellulose and lignin. This indicates that the three main components are connected through homeostatic regulatory mechanisms. This cross-component interaction has significant implications: modifying one barrier (e.g., reducing cellulose crystallinity) may inadvertently increase another (e.g., increasing lignin encapsulation) if compensatory pathways are activated. Therefore, effective genetic improvement strategies must consider the cell wall as an integrated system, rather than targeting individual components in isolation.

**Figure 2:**
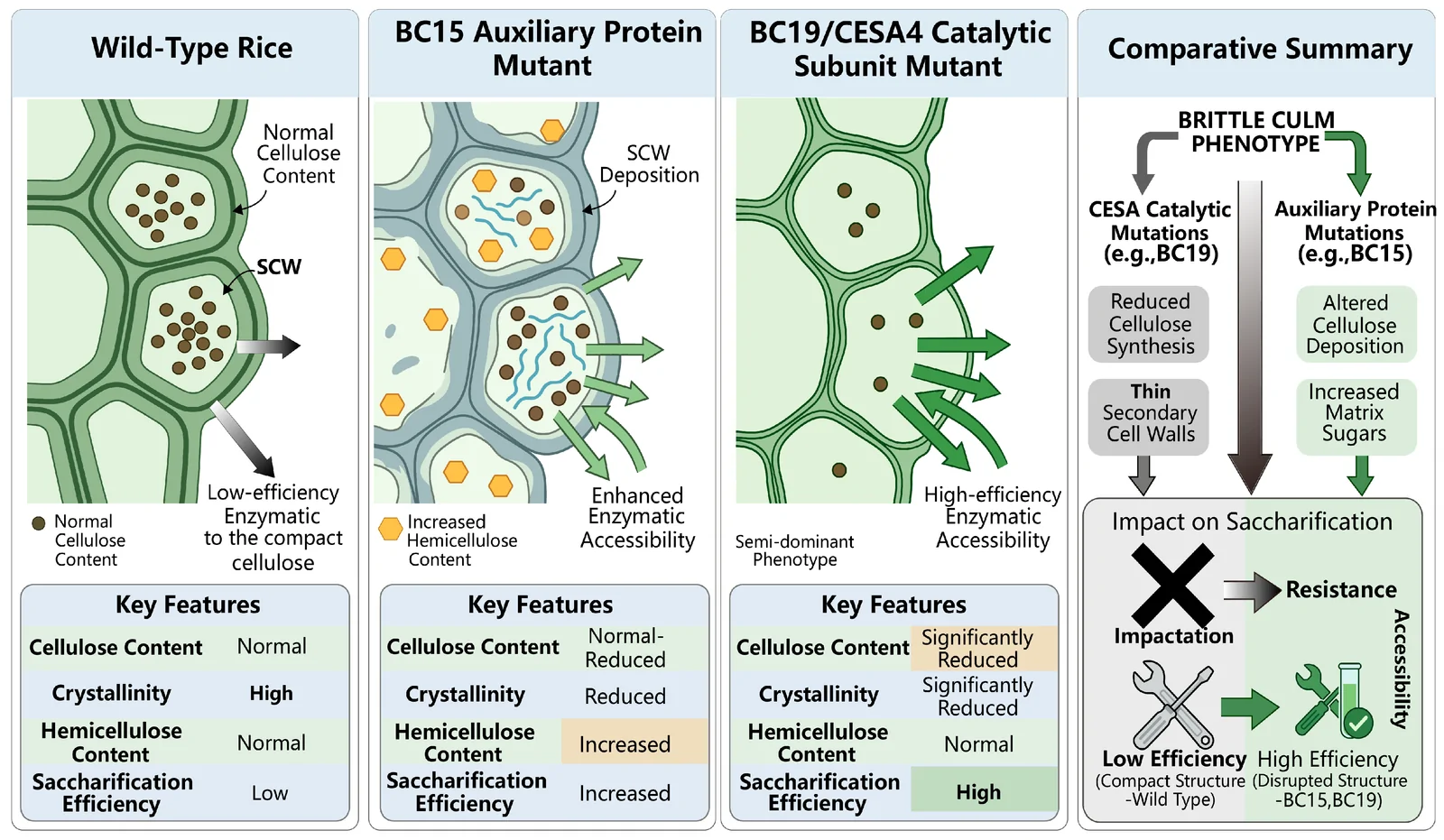
Comparison of brittle culm mutant types and cell wall structures in rice. Brittle culm mutants are classified into two categories based on their gene function and mutation type. CESA catalytic subunit mutants: loss-of-function alleles (e.g., *OsCESA4*, *OsCESA7*, and *OsCESA9* null mutants) severely reduce cellulose content and secondary wall thickness, leading to brittle phenotypes with severe growth defects. Semi-dominant alleles, including *BC19*/*P507S* (*OsCESA4*) and *D387N* (*OsCESA9*), partially reduce cellulose crystallinity and deposition while maintaining normal plant morphology and grain yield. The *FC17*/*CESA4* mutation further induces compensatory increases in hemicellulose and lignin, increasing saccharification yield by 1.68-fold and improving dry matter digestibility from 26.7% to 31.4%. Non-catalytic subunit mutants: *BC15* (*OsCTL1*), encoding a Golgi-localized chitinase-like protein, affects cellulose microfibril arrangement rather than catalytic synthesis. The bc15 mutant shows lower NDF and ADF contents, higher water-soluble carbohydrates, and faster initial ruminal fermentation rates, though with a context-dependent degradation pattern. The *CEF1*/*OsMYB103L* transcription factor links cell wall regulation to degradability by directly binding to *CESA4*, *CESA7*, *CESA9*, and *BC1* promoters.

Semi-dominant *CESA* alleles have emerged as particularly valuable genetic resources. Ma et al. (2021) identified a unique semi-dominant brittle culm mutant, *BC19* [11]. This mutant harbors a C-to-T single nucleotide substitution in *OsCESA4*, resulting in a *P507S* missense mutation. Unlike null mutants, the *BC19*/*P507S* heterozygote exhibited a distinct brittle culm phenotype but maintained normal plant morphology and grain yield (Figure 2). Furthermore, the cellulose content was reduced by approximately 40%, and crystallinity index significantly decreased. This semi-dominant characteristic is particularly valuable for breeding, as heterozygous plants can achieve enhanced straw degradability without significant yield penalty.

The *OsCESA9 D387N* semi-dominant mutation (*sdbc1*) provides a particularly compelling case. The D387N mutant protein competitively binds to OsCESA4 and OsCESA7, forming partially non-functional cellulose synthase complexes [12]. This interaction leads to a reduction in cellulose deposition and secondary wall thickness. Notably, heterozygous sdbc1 plants maintained normal lodging resistance and grain yield, while the altered cell wall properties improved enzymatic saccharification efficiency and enhanced salt stress tolerance, exemplifying a rare synergism between quality and stress tolerance. Importantly, the mutant straw was described as being easily crushed by harvesters and more susceptible to degradation, providing direct evidence linking *CESA* alleles to field straw decay traits. Furthermore, the conserved-site mutation in *OsCESA9* enhances lodging resistance, suggesting that the relationship between cell wall modification and mechanical strength is allele-dependent and context-specific [13].

*BC15* (*OsCTL1*) encodes a membrane-associated chitinase-like protein that is localized to the Golgi apparatus. Its function relates to cellulose microfibril deposition and arrangement rather than catalytic synthesis. The *bc15* mutation leads to reduced cellulose content, increased hemicellulose levels, and brittle culm phenotypes [14]. Yi et al. (2022) conducted a systematic investigation into the effects of the *BC15* mutation on in vitro ruminal fermentation, revealing a context-dependent degradation pattern [15]. Specifically, *bc15* mutant straw exhibited significantly lower neutral detergent fiber (NDF) and acid detergent fiber (ADF) contents, along with higher levels of water-soluble carbohydrates and neutral detergent solutes, resulting in accelerated initial degradation rates within the first 12 h of fermentation. However, the cumulative methane production over 48-h was lower than that of the wild type, suggesting that the altered carbohydrate composition favors initial degradation, and it may not optimally support sustained methanogenesis (Figure 2). This context-dependent degradation effect highlights the importance of defining the endpoint of degradation—whether for biofuel production, ruminant feed, or field return—when evaluating mutant performance.

Various types of brittle culm mutants exhibit significant differences in cell wall structural modifications, as illustrated in Figure 2. The transcription factor CEF1/OsMYB103L provides a critical link between cell wall regulation and degradability. Through map-based cloning and biochemical validation, CEF1 directly interacts with the promoters of *CESA4*, *CESA7*, *CESA9*, and *BC1*, thereby orchestrating cellulose deposition [16]. This suggests that upstream regulatory genes may offer more comprehensive control over cell wall properties compared to targeting individual structural genes. This concept will be further examined in relation to the regulation of lignin biosynthesis regulation.

## 3. Lignin Biosynthesis and Cell Wall Modification Genes

The lignin biosynthesis pathway constitutes the fundamental genetic framework governing the degradability of rice straw. Initiating from phenylalanine, this pathway proceeds through deamination, hydroxylation, methylation, reduction, and polymerization, culminating in the production of H, G, and S lignin monomers. A total of 91 lignin biosynthesis gene family members were identified in rice through genome-wide analysis, noting a significant expansion in the *PAL*, *CCoAOMT*, *CCR*, and *CAD* families, attributed to tandem and segmental duplication events [5]. This section examines the pivotal enzyme genes, their transcriptional regulation, and the closely related hemicellulose modification genes that collectively determine cell wall recalcitrance.

### 3.1. Lignin Biosynthesis Enzyme Genes: From Pathway Suppression to Structural Engineering

**Figure 3:**
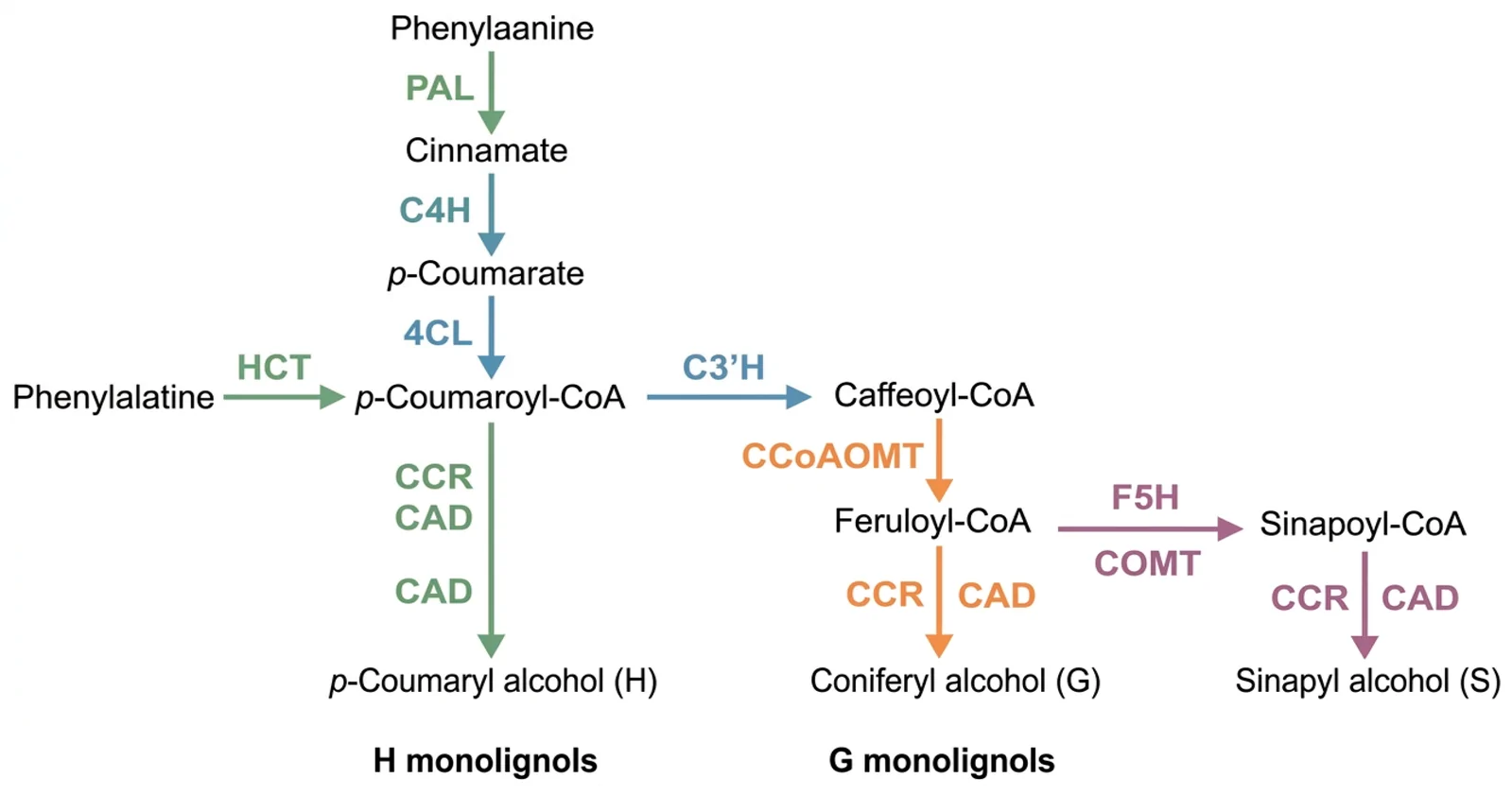
Lignin biosynthesis pathway in rice. The pathway proceeds from phenylalanine through sequential reactions (deamination, hydroxylation, methylation, reduction, and polymerization) to generate H, G, and S lignin monomers. Key enzymes and their regulatory levels are indicated: upstream genes (*PAL*, *C4H*, and *4CL*) control total carbon flux allocation, whose suppression causes severe growth defects; midstream genes (*OsC3′H1* and *Os4CL3*/*5*) regulate branch-point metabolic flux toward H-type or G/S-type lignin; downstream genes (*OsCAD2*, *OsCOMT*, and *OsFNSII*) modify monomer composition and structure, offering the most promising targets for structural engineering. The *OsCAD2* site-directed modification alters H/G ratios and increases saccharification yield by 61–72% without severe growth penalties. *OsCOMT* suppression reduces S-unit content and increases cell wall porosity by up to 1.8-fold. *OsFNSII* loss eliminates tricin nucleation sites, reducing steric hindrance. Beyond the canonical pathway, the SHP-GLOX system introduces in situ lignin degradation capacity through a senescence-induced promoter, achieving up to 30% lignin reduction at maturity. Green arrows denote the H-monolignol branch (p-coumaryl alcohol synthesis), blue arrows denote early phenylpropanoid steps and G-branch initiation, orange/brown arrows denote the G-monolignol branch (coniferyl alcohol synthesis), and purple arrows denote the S-monolignol branch (sinapyl alcohol synthesis).

Enzymes involved in the lignin biosynthesis pathway exhibit varying degrees of influence on the total lignin content, composition, and degradability. Early pathway genes (e.g., *PAL* and *C4H*) typically cause significant lignin reduction when suppressed, but with severe growth defects. Complete knockout of these genes can be lethal [17] (Figure 3). Simultaneous suppression of *C4H* and *4CL* in rice significantly decreased lignin content in mature straw through RNA interference (RNAi) [18]. However, while the suppression of C4H alone did not cause obvious agronomic abnormalities, complete pathway blockade risked embryonic lethality, especially in backgrounds where *C3′H* was knocked out.

*OsC3′H1* (p-coumaroyl ester 3-hydroxylase) serves as a pivotal regulatory node within the phenylpropanoid pathway, determining metabolic flux towards either H-type or G/S-type lignin biosynthesis (Figure 3). Partial knockdown of *OsC3′H1* by RNAi increased H-unit proportions while maintaining normal plant growth. In contrast, a complete knockout achieved through CRISPR/Cas9 caused severe developmental abnormalities, including growth arrest, irregular vascular bundles, and ectopic lignification, although metabolic redundancy allows some G/S synthesis [19]. These findings highlight the importance of precise expression control rather than complete gene knockout for maintaining normal plant development.

*OsCAD2* currently represents the most compelling candidate for precise lignin structural engineering. CRISPR/Cas9 site-directed modification of *OsCAD2* resulted in a 34% reduction in H-units and a 16% increase in G-units, with total lignin decrease of only 15–16% [20]. Critically, the mutation caused incorporation of atypical hydroxycinnamaldehydes into the lignin polymer, resulting in a 1.8-fold increase in cell wall porosity. Under mild alkaline pretreatment, the modified lignin extraction rate reached approximately 70%, and hexose yield improved 61–72%, marking the highest reported improvement for lignin-altered rice lines. Further analyses revealed that *OsCAD2* mutant lignin formed smaller nanoparticles (1.5–1.9-fold reduction), and the mutant seedlings showed enhanced cadmium accumulation capacity, suggesting potential applications in phytoremediation [21] (Figure 3). These findings demonstrate that lignin structure and chemical depolymerizability may be more important than total lignin content for improving degradability.

The *OsCOMT* gene family has been comprehensively characterized. Rice contains 33 genes with methyltransferase-2 domains [22]. CRISPR/Cas9-mediated suppression of *OsCOMT* reduces S-unit content, increases cell wall porosity by up to 1.8-fold, and improves enzymatic saccharification efficiency by 20–26% [21]. However, a complete knockout of *OsCOMT* is potentially lethal, and RNAi-mediated partial suppression does not significantly affect agronomic traits, underscoring the importance of precise expression control. *OsCAldOMT1* (*OsCOMT1*) functions as a bifunctional O-methyltransferase involved the in synthesis of both S-lignin and tricin-lignin [23] (Figure 3). The CRISPR-mediated knockout of *OsCAldOMT1* completely eliminates S-units and tricin from lignin, incorporating 5-hydroxyguaiacyl units instead. Analyses using solid-state nuclear magnetic resonance (ssNMR) and X-ray diffraction (XRD) have revealed that *OsCAldOMT1* loss severely disrupts cellulose lattice ordering and dramatically increases molecular mobility of cellulose. This alteration results in a highly relaxed lignocellulose assembly with extensively exposed cellulase binding sites, an effect that is even more pronounced than *OsCAD2* mutation.

OsFNSII (flavone synthase II) provides an unexpected connection between flavone and lignin metabolism. The loss of OsFNSII function completely eliminates tricin from lignin, thereby reducing steric hindrance and improving saccharification rates. This demonstrates that targeting non-canonical lignin components, such as tricin nucleation sites, can be an effective strategy for improving degradability without directly modifying lignin biosynthesis [23]. Furthermore, *OsCAld5H1*/*CYP84A5* plays a crucial role in regulating the S/G composition of lignin: its downregulation shifts lignin composition towards G-units, while its upregulation shifts it toward S-units. This gene is primarily expressed in lignifying vegetative tissues [24]. These insights support the strategy of targeting monomer composition and bond type rather than relying solely on the acid-insoluble lignin percentage as the screening criterion.

The lignin biosynthesis pathway constitutes a linear and branched regulatory network extending from phenylalanine to three lignin monomers (Figure 3). The regulatory dimensions can be classified into three levels: upstream genes (*PAL*, *C4H*, and *4CL*) that govern the allocation of total carbon flux; midstream genes (*HCT*, *C3′H*, and *CCoAOMT*) that determine monomer type partitioning; and downstream genes (*CCR*, *F5H*, *CAD*, and *COMT*) that catalyze the final steps of monomer synthesis, thereby directly determining the S/G ratio and the structural composition of lignin. It is important to distinguish between the two fundamentally different strategies of improving degradability through the lignin pathway: reducing the total amount of lignin or modifying its composition or polymer structure. Suppressing upstream genes (*PAL*, *C4H* and *4CL*) primarily reduces total lignin content, but this typically causes severe growth penalties as lignin is essential for vascular function and structural integrity. In contrast, manipulating downstream genes (*OsCAD2*, *OsCOMT* and *OsFNSII*) alters the composition of the monomers, the types of bonds and the polymer structure, while preserving most of the lignin. For instance, modifying *OsCAD2* does not substantially reduce total lignin content (by only 15–16%), but it does change the H/G ratio and introduce atypical hydroxycinnamaldehydes, thereby increasing saccharification by 61–72%. Similarly, *OsCOMT* suppression shifts the S/G ratio rather than eliminating lignin. These findings demonstrate that lignin structure and chemical depolymerisability are more important determinants of degradability than total lignin content, and that downstream structural engineering is a more viable breeding strategy than upstream pathway suppression (Table 2).

### 3.2. The Shp-Glox System: In Situ Lignin Degradation

An alternative approach to modifying biosynthetic genes involves the introduction of targeted degradation mechanisms. A streptavidin-hook protein (SHP) fused with glyoxal oxidase (GLOX) was constructed to anchor in the cell wall and degrades lignin in situ [25]. This system operates on a synergistic logic: GLOX catalyzes the oxidation of glyoxal and methylglyoxal to produce H_2_O_2_, which is subsequently utilized by SHP (a class III plant peroxidase from soybean seed coat) to oxidatively degrade phenolic compounds and lignin. When expressed in transgenic rice under a senescence-induced promoter, this system demonstrated that, at the flowering stage, lignin content in certain transgenic lines was comparable to or slightly exceeded that of wild-type plants. However, at maturity, nearly all transgenic lines showed significantly lower lignin content, with reductions reaching up to 30%. Furthermore, enzymatic saccharification and simultaneous saccharification fermentation (SSF) both produced significantly higher reducing sugar and ethanol yields than wild type. Critically, no negative impact was observed on agronomic traits including plant height, panicle length, effective tiller number, grains per panicle, seed-setting rate, or thousand-grain weight. The senescence-induced expression strategy ensures that lignin degradation occurs only after grain filling is complete, avoiding any interference with normal plant development.

### 3.3. Hemicellulose Modification Genes: Side-Chain Fine-Tuning

Hemicellulose modification genes have received less attention than cellulose and lignin, yet they constitute a crucial regulatory aspect with relatively minor impacts on plant growth and development. The arabinoxylan side-chain modification system involves several key genes with distinct mechanisms.

Among these, *OsAT10*, encoding a BAHD acyltransferase, has been identified as a pivotal gene responsible for the p-coumaroylation of arabinoxylan. Overexpression of *OsAT10* dramatically increases p-coumaric acid (pCA) content and decreases ferulic acid (FA) content. The reason is inhibition of *OsAT9*-mediated feruloylation due to substrate competition for UDP-arabinose, resulting in improved saccharification [26]. This effect has been replicated in heterologous systems; *OsAT10* overexpression in sorghum and switchgrass similarly increased pCA, decreased FA, and improved saccharification by up to 28%, with an unexpected reduction in total lignin in sorghum. However, CRISPR/Cas9-mediated knockout of *OsAT10* specifically removes pCA without changing saccharification, demonstrating that pCA itself does not directly cause recalcitrance [27]. This pCA paradox reveals that the observed improvement from *OsAT10* overexpression actually stems from reduced ferulate cross-linking, achieved through metabolic flux redirection. This improvement is not due to pCA accumulation per se. Such a distinction provides a critical insight for designing genetic modification strategies.

The *OsHCALDH2*/3 genes, encoding aldehyde dehydrogenases, participate directly in ferulate metabolism. CRISPR/Cas9 editing of these genes reduces ferulate ester content by approximately 45%, effectively cutting the cross-linking network that restricts enzyme access to cellulose [27]. The *BS1* gene, encoding a GDSL esterase, participates in xylan O-2/O-3 deacetylation. The *bs1* mutant revealed the importance of xylan acetylation levels for secondary wall formation and patterning; the acetylation state may alter xylan interactions with cellulose and lignin, and may also affect the ability of hydrolytic enzymes to access substrates [28]. These findings suggest that fine-tuning side-chain modifications, such as reducing ferulate cross-linking and adjusting acetylation, may be beneficial. The reason is preferable to disrupt backbone synthesis, as mutations in backbone genes such as *OsXAX1* (*GT61*) and *OsIRX10* (*GT47*) improve saccharification but are accompanied by growth defects.

### 3.4. Transcription Factor Networks: The Master Switches

Lignin and cell wall synthesis gene expression is regulated by a hierarchical transcription factor network. Within this network, NAC and MYB transcription factors constitute the key regulatory cascade, whose NAC factors serve as master switches at the top of the hierarchy.

Specifically, *OsSWN1* and *OsSWN2* are distinct *NAC* regulators exhibiting critical tissue-specific functions. *OsSWN2* is specifically expressed in xylem vessels, and its promoter-driven expression of the chimeric repressor *OsSWN2S*. This expression can cause vessel collapse and wilting even under normal water supply. In contrast, *OsSWN1* is expressed in sclerenchyma fibers, and its promoter-driven expression of the same repressor preserves vessel function while reducing sclerenchyma wall thickness, as well as lignin and xylan contents. This modification improves degradability without growth inhibition [29]. This tissue-specific strategy provides a critical approach for avoiding the developmental trade-offs associated with global lignin suppression.

The downstream cascade involves OsSND2, OsNAC29, and OsNAC31, which bind to the *OsMYB61* promoter at SNBE sites to activate its expression. OsMYB61 then directly binds to *OsCESA* family promoters to coordinate cellulose deposition. *RRS1* acts as a negative regulator of stem strength, and OsbHLH (OsICE1) interacts with OsNAC31 for fine-tuning [29].

*RLM1* (*LOC_Os05g46610*), an R2R3-MYB transcription factor, binds to the OsCAD2 promoter. The interacting protein OsMAPK10 of RLM1 positively regulates leaf and stem lignin content. This interaction establishes a MAPK–MYB–OsCAD2 regulatory network for secondary wall formation. Notably, *RLM1* knockdown materials showed yield increases exceeding 11% without significant agronomic costs, though the primary phenotype studied was leaf morphology and secondary wall development rather than the decomposition of mature straw in the field [30].

The *DEP1* locus provides another example of a ‘multiple trait–cell wall–saccharification’ relationship. The *dep1* editing materials showed enhanced stem mechanical properties; increased cellulose, hemicellulose, and pectin contents; decreased lignin content and cellulose crystallinity; and improved enzymatic saccharification after alkaline pretreatment [31]. This suggests that cell wall regulation can simultaneously affect lodging resistance and biomass utilization. However, it also underscores the potential tension between lodging resistance and enzymatic saccharification, requiring clear definition of the target endpoint.

## 4. Genetic Mapping, Gene Editing, and Breeding Strategies

### 4.1. Qtl and Gwas: Identifying Genetic Architecture of Straw Degradability

Straw degradability is a complex quantitative trait controlled by multiple genes. The first quantitative trait loci (QTL) mapping study for stem saccharification digestibility in rice identified multiple QTL on chromosomes 1, 6, 7, and 11 [32]. Subsequently, eight QTL clusters that simultaneously control lignin monomer content and biomass digestibility were identified, with four QTL affecting both lignin composition and saccharification [33]. A critical finding from this study was that favorable alleles can be pyramided from these QTL. Total lignin content remained comparable, whereas sugar release was significantly improved. Importantly, this improvement incurred no penalty to lodging resistance. Moreover, the absolute content of lignin monomers was found to be negatively correlated with saccharification, while the H-unit relative proportion and H/G ratio had positive effects. This finding shifts the focus of the candidate gene priority from total lignin reduction to monomer composition optimization.

Genome-wide association studies (GWAS) leverage historical recombination events in natural populations to achieve gene-level resolution. A total of 64 QTL and 173 significant SNPs for lignocellulosic compounds and fermentable sugar content were identified using 33,484 single nucleotide polymorphism (SNP) markers on 149 rice accessions [34]. They identified 21 candidate genes distributed across five major networks: cytoskeletal dynamics and wall precursor transport, plant growth and development, direct cell wall biosynthesis, cell wall modification enzymes, and transcriptional regulation (*WRKY*/*ERF*/*MYB* as primary factors). Notably, gene modules related to the dry weight of panicle and third internode showed the highest positive correlations with overall biomass (r = 0.64 and 0.57, respectively). It is important to note that transcriptomic and expression analyses alone cannot establish causal relationships between candidate genes and straw degradability. Differences in expression may reflect the consequences, rather than the cause, of cell wall modification, and many genes identified through expression profiling remain unvalidated at the functional level.

The first GWAS specifically for rice straw digestibility identified QTL and candidate genes including *OsAT10*, *OsIRX9*, and *OsMYB58*/*63-L* [35]. These genes have been reproducibly associated with straw digestibility across multiple studies, representing priority breeding targets. *OsAT10* occupies a shared QTL region for both digestibility and silica content. This region exhibits contrasting bi-allelic effects between sugar release and silica accumulation [36]. A genome-wide association study on rice straw cell wall components identified marker-trait associations, with lignin content GWAS markers including *4CL3*, *WRKY71*, and *OsACS7*, further expanding the catalog of candidate genes [37]. However, numerous newly identified candidates, such as *GH16*, *CSLD2*, and *WAK12*, remain functionally uncharacterized for straw degradability. This highlights a broader issue: many candidate genes identified through GWAS lack functional validation beyond expression correlations. An increase in the expression of a gene during straw degradation or senescence does not demonstrate that the gene directly contributes to degradation. A rigorous hierarchy of evidence should distinguish between genes that have been validated through mutant analysis, CRISPR editing or transgenic complementation, and those that are only supported by expression data or QTL co-localisation. Table 3 classifies the current level of evidence for each major gene discussed in this review.

The silica content has emerged as an underexplored but significant factor in straw degradability. Multiple studies have confirmed a strong negative correlation between silica content and digestibility. GWAS have shown that major silica MTAs co-localize with silicon uptake genes *OsOPT2*, *OsOPT3*, and *OsUCP2*. Haplotype analysis also showed that superior alleles were associated with up to 15.9% lower silica content [37,38]. Additionally, *OsSEX4* encodes a chloroplast-localized glucan phosphatase rather than a cell wall structural protein. *OsSEX4* knockdown causes excess starch accumulation in straw, producing 50% more bioethanol than wild type, expanding the range of genetic targets beyond cell wall biosynthesis [39].

### 4.2. Crispr/cas9 Strategies and the Trade-Off Between Degradability and Agronomic Performance

CRISPR/Cas9 gene editing technology has made precise improvement of straw degradability possible. Currently reported editing targets encompass three major dimensions of cell wall synthesis: hemicellulose modification (*UXE* and *XAT*), lignin biosynthesis (*OsCAD2*, *Os4CL3*/*5*, *OsCOMT*, and *OsHCALDH2*/*3*), and cellulose synthesis (*CESA* regulatory regions). synergistic effects from simultaneous editing of *UXE* and *XAT* can achieve greater reductions in cellulose crystallinity and improvements in saccharification than single-gene knockouts [40]. The *OsCAD2* case exemplifies the advantage of precision modification. Rather than completely knocking out the gene, site-directed modification preserved partial enzymatic activity. This modification altered the monomer composition of the resulting lignin, which in turn substantially improved saccharification without severe growth penalties [20].

The core challenge in genetic improvement of straw degradability is balancing reduced cell wall recalcitrance with maintained normal growth, lodging resistance, and yield. The ideal strategy is not extreme weakening of the cell wall, but selection of mild allelic variants that can alter crystallinity, monomer composition, cross-linking patterns, or tissue distribution. This strategy aims to preferentially achieve expression differences in mature stems, non-grain tissues, or late senescence stages. Several strategies have emerged to address this trade-off.

First, semi-dominant alleles offer a unique approach. For example, the *BC19*/*P507S* heterozygote maintains normal plant morphology and grain yield despite approximately 40% reduction in cellulose content [11]. Similarly, the *OsCESA9 D387N* heterozygous plants improve both salt tolerance and straw degradability while maintaining yield [12]. Second, tissue-specific promoters can restrict gene editing effects to specific tissues and developmental stages. In particular, the *OsSWN1* sclerenchyma-specific promoter and senescence-induced promoters avoid interference with primary cell wall function [29,41]. Third, multi-gene coordinated editing may achieve the goal of dual-purpose rice cultivars. This strategy progresses through three steps: cell wall cross-linking is first reduced through hemicellulose modification genes, lignin monomer composition is then optimized through pathway genes, and agronomic performance is finally maintained through semi-dominant *CESA* alleles.

A critical caveat must be emphasized: different degradability endpoints require different evidentiary foundations. Enzymatic saccharification following alkaline pretreatment is relevant for biofuel production, in vitro ruminal digestibility pertains to feed quality, and in situ field decomposition is relevant for straw return to fields. These endpoints share some candidate genes but they require unique evidence interpretation. The microbial inoculation can improve straw decomposition rates by up to 4.9% with simultaneous rice yield increases of 790 kg/ha, by reshaping the structure and function of lignocellulose-degrading microbial consortia [7]. More broadly, the decomposition of straw in the field involves a complex consortium of bacteria, fungi and actinomycetes, the composition of which is shaped by both the chemistry of the straw and the conditions of the soil. Studies have shown that lignin-rich straw favors basidiomycete fungi, whereas cellulose-enriched straw favors ascomycete-dominated communities. The interaction between rice genotype and microbial ecology is a critical yet underexplored dimension of straw degradability that requires systematic investigation. This confirms that rice genotype provides the substrate accessibility background while actual decomposition is driven by microbial ecology, and genotype effects must be validated within the appropriate microbial context. The causal chain from gene editing to cell wall chemistry and structure, to enzyme and microbial accessibility, to sugar release or field mass loss, must be established through direct measurement rather than inferred from expression data alone. It must be acknowledged that there is currently very little field-level validation of gene-edited or mutant rice lines for straw degradability. Most of the functional studies cited in this review were conducted in greenhouses or in controlled environments. The *OsCESA9 D387N* mutant [12] and the *SHP*-*GLOX* transgenic lines [25] are rare examples of lines for which field straw decay traits have been explicitly evaluated; however, neither has been tested across multiple field environments or growing seasons. The discrepancy between improvements demonstrated in the laboratory and verified performance in the field is one of the most significant barriers to translating genetic discoveries into breeding applications and should be prioritized in future research.

## 5. Conclusions and Perspectives

Genetic improvement of rice straw degradability has progressed from single-gene functional verification to systems-level regulation. Under the three-parameter framework of ‘cell wall composition–synthesis genes–degradation efficiency,’ this review has synthesized the regulatory mechanisms by which rice endogenous genes influence straw degradability, yielding the following major conclusions:

Cellulose synthase genes (*CESA* family) play a crucial role in determining cellulose crystallinity and microfibril arrangement, constituting the foundational genetic factors affecting straw degradability. Semi-dominant *CESA* mutants (e.g., *BC19*/*P507S*, *OsCESA9*, and *D387N*) can effectively reduce cell wall recalcitrance without significantly affecting agronomic traits, thus providing practical allelic resources for heterozygous breeding strategies [11,12]. Modifications to the cell wall initiate systemic reprogramming accompanied by compensatory changes, highlighting the necessity for approaches at the systems level [9]. In parallel, the genes involved in the lignin biosynthesis pathway form a linear and branched regulatory network. Within this network, downstream genes, particularly *OsCAD2*, offer the most promising targets for structural engineering rather than total content reduction. The *OsCAD2* case achieves 61–72% saccharification improvement through monomer composition alteration. It demonstrates that lignin structural modification is more effective than total content reduction [20]. The SHP-GLOX system represents a fundamentally different approach by introducing in situ lignin degradation capacity [25].

At the fine-tuning level, genes responsible for modifying hemicellulose side chains, such as *UXE*, *XAT*, *BAHD* acyltransferases, and *OsHCALDH2*/*3*, regulate arabinoxylan side-chain substitution and ferulate cross-linking. This regulation forms a complementary regulatory dimension with relatively minor impacts on plant growth [4,40]. The ‘pCA paradox’ revealed by *OsAT10* studies demonstrates that metabolic flux redirection can be an effective and non-obvious strategy for improving degradability, rather than direct biochemical product effects [26]. This finding has broader implications: it suggests that genes whose overexpression does not directly affect a known cell wall component can still enhance degradability by indirectly altering metabolic pathways. Non-obvious genetic targets of this kind could be systematically identified by combining metabolomic profiling with saccharification phenotyping. The pCA paradox also cautions against equating the accumulation of a single cell wall component with a predictable outcome in terms of degradation, thereby reinforcing the need for multi-parameter evaluation frameworks. At a higher regulatory level, transcription factor networks, particularly the NAC-MYB cascade, function as master switches for coordinating cell wall biosynthesis. Tissue-specific promoters, such as *OsSWN1*, and regulatory hubs like *RLM1* and *DEP1*, offer strategies for simultaneously improving degradability and agronomic performance [29,30,31]. However, the potential tension between lodging resistance and natural degradation requires clear definition of target endpoints.

In summary, several important research gaps remain. Firstly, most gene functional studies were conducted using a single genetic background, such as Nipponbare or Zhonghua 11, which limits the systematic validation across different genetic backgrounds [32,37]. Secondly, the definition of ‘degradability’ is not unified. Enzymatic saccharification, in vitro ruminal digestibility, and in situ field decomposition are distinct endpoints that cannot be directly compared or conflated [6,15]. Thirdly, reports on CRISPR editing aimed at enhancing straw degradability remain limited, with insufficient off-target control and multi-environment validation [20,27]. Looking ahead, the establishment of a unified end-phenotype system and a closed-loop validation platform, incorporating NIRS, GWAS, stem transcriptomics, and targeted editing, is needed. This platform will be essential for translating genetic discoveries into breeding applications. The integration of genomic selection with gene editing technology is expected to accelerate the development of dual-purpose rice cultivars, thereby contributing to the circular bioeconomy and sustainable agriculture.

## Figures and Tables

**Table 1:** Structural characteristics and degradation barriers of three major rice straw cell wall components.

Component	Structural Features	Degradation Barrier Mechanism	Representative Genes
Cellulose	High-crystallinity β-1,4-glucan microfibrils	Restricts enzyme accessibility, reduces hydrolysis rate	*OsCESA4*/*7*/*9* [8]
Hemicellulose	Arabinoxylan with ferulate ester cross-links	Forms dense encapsulating network, impedes enzyme diffusion	*UXE*, *XAT*, *BAHD* [4]
Lignin	H-G-S ternary composition, LCC complexes	Non-specific enzyme adsorption, physical encapsulation of polysaccharides	*PAL*, *4CL*, *CAD*, *COMT* [5]

**Table 2:** Functional verification and phenotypic effects of key lignin biosynthesis enzyme genes in rice.

Gene	Encoded Enzyme	Mutant/Editing Effect	Reference
*OsPAL*	Phenylalanine ammonia-lyase	Total lignin decreased; growth and developmental defects	[17]
*Os4CL3*	4-Coumarate:CoA ligase	Total lignin significantly decreased	[5]
*Os4CL5*	4-Coumarate:CoA ligase	Lignin monomer ratio altered	[5]
*OsCAD2*	Cinnamyl alcohol dehydrogenase	G-units increased by 16%, H-units altered, saccharification yield increased by 61–72%	[20]
*OsCOMT*	Caffeic acid O-methyltransferase	S/G ratio altered, porosity increased by 1.8-fold, saccharification improved by 20–26%	[5,21]

**Table 3:** Summary of Gene Effects on Rice Straw Degradability and Corresponding Experimental Conditions.

Gene	Modification Type	Key Effect on Degradability	Magnitude of Improvement	Experimental Conditions	Evidence Level
*OsCESA4* (*FC17*)	Loss-of-function mutation	Reduced cellulose crystallinity, increased saccharification	1.68-fold increase in saccharification	Greenhouse-grown, alkaline pretreatment	Mutant validation
*OsCESA4* (*BC19*/*P507S*)	Semi-dominant allele	Reduced cellulose, normal yield	~40% cellulose reduction, no yield penalty	Field-grown	Mutant validation
*OsCESA9* (*D387N*)	Semi-dominant allele	Improved salt tolerance and degradability	Normal yield maintained	Field-grown	Mutant validation
*OsCAD2*	CRISPR site-directed modification	Altered H/G ratio, increased porosity	61–72% saccharification improvement, 1.8-fold porosity	Greenhouse, alkaline pretreatment	CRISPR validation
*OsCOMT*	CRISPR/RNAi suppression	Reduced S-units, increased porosity	20–26% saccharification improvement, 1.8-fold porosity	Greenhouse	CRISPR/RNAi validation
*OsCAldOMT1*	CRISPR knockout	Eliminated S-units and tricin, disrupted cellulose ordering	Pronounced cellulase binding site exposure	Greenhouse	CRISPR validation
*OsFNSII*	Loss-of-function	Eliminated tricin from lignin	Reduced steric hindrance, improved saccharification	Greenhouse	Mutant validation
*OsAT10*	Overexpression	Increased pCA, decreased FA	Improved saccharification	Greenhouse	Overexpression validation
*OsHCALDH2*/*3*	CRISPR editing	Reduced ferulate esters	~45% reduction in ferulate esters	Greenhouse	CRISPR validation
*SHP-GLOX*	Transgenic system	In situ lignin degradation	Up to 30% lignin reduction	Greenhouse	Transgenic validation
*OsSEX4*	Knockdown	Excess starch in straw	50% more bioethanol	Greenhouse	Knockdown validation

## Data Availability

No new data were created or analyzed in this study. Data sharing is not applicable to this article.

## Abbreviations

The following abbreviations are used in this manuscript: ADF, Acid Detergent Fiber; AX, Arabinoxylan; BAHD, BEAT, AHCT, HCBT, and DAM1 (acyltransferase family); CrI, Crystallinity Index; CSC, Cellulose Synthase Complex; CESA, Cellulose Synthase A; FA, Ferulic Acid; GWAS, Genome-Wide Association Study; LCC, Lignin–Carbohydrate Complex; NDF, Neutral Detergent Fiber; pCA, p-Coumaric Acid; QTL, Quantitative Trait Loci; RNAi, RNA Interference; SNBE, Secondary Wall NAC Binding Element; ssNMR, Solid-State Nuclear Magnetic Resonance; XRD, X-Ray Diffraction.
